# P64. T cell re-direction against Glypican-3 for immunotherapy of hepatocellular carcinoma

**DOI:** 10.1186/2051-1426-2-S2-P38

**Published:** 2014-03-12

**Authors:** C Dargel, M Bassani-Sternberg, K Krebs, S Wilde, D Schendel, DH Busch, M Mann, U Protzer

**Affiliations:** 1 Institute of Virology, Technische Universität, München, Germany; 2 Institute of Biochemistry, Max-Plank-Institute, München, Germany; 3 Institute of Molecular Immunology, Helmholtz Zentrum, München, Germany; 4 Institute for Medical Microbiology Immunology and Hygiene, Technische Universität, München, Germany

## 

Hepatocellular carcinoma (HCC) is the third most common cause of cancer related mortality world-wide and therapeutic options are very limited. A new therapeutic approach is the adoptive T cell therapy of HCC. Glypican-3 (GPC3) as a tumour associated antigen is expressed in up to 60% of all HCC but not in healthy human liver tissue. Therefore, our goal is to generate cytotoxic T lymphocytes (CTL), which are capable of recognizing and eliminating GPC3-expressing tumor cells.

Immunodominant epitopes for GPC3 have not been described yet. In this study we used Ultra Nano HPLC coupled on-line to the Q Exactive mass spectrometer to obtain a comprehensive HLA class I peptidome from a GPC3 and HLA-A2 positive hepatoma cell line. The resulting data were analysed using the MaxQuant bioinformatics platform. Two HLA-A2 bound GPC3 peptides could be identified, later on referred to as GPC3-P1 and GPC3-P2. These results enable us to target GPC3 epitopes that are presented on GPC3 positive HCC cells.

To isolate tumour reactive high avidity T cells, an allo-restricted stimulation approach was used. For stimulation of naïve T cells, autologous dendritic cells were co-transfected with GPC3 and HLA-A2 RNA and used as antigen presenting cells. T cells from the naïve T cell repertoire of HLA-A2 negative donors were co-cultured with and expanded on these HLA-A2+ GPC3+ DCs. After two weeks, MHC streptamer-positive CD8^+^ T cells specific for both targeted GPC3 epitopes were detected (<1%). We were able to enrich these cell populations further to 35% GPC3-P1- and 57% GPC3-P2-MHC streptamer-positive T cell lines and grew T cell clones from them. In a co-culture with GPC3-P1/ -P2 peptide loaded T2 cells we identified T cell clones displaying specific effector function by IFNγ secretion. Functional T cell clones showed strong GPC3 MHC streptamer binding.

We have cloned the first T cell receptors (TCR) to either GPC3 peptide from these T cell clones. T cells engrafted with the GPC3 specific TCRs showed strong GPC3 MHC streptamer binding. When co-cultured with GPC3 peptide loaded target cells or a GPC3 expressing hepatoma cell line (HepG2), GPC3 TCR transduced T cells secreted IFNγ. Furthermore cytotoxicity was observed by killing of up to 60% of HepG2 cells. GPC3-directed T cell therapy shows great promise for the treatment of HCC.

